# GP perspectives on genomics in primary care: a qualitative study on using polygenic risk scores to evaluate cancer risk

**DOI:** 10.3399/BJGP.2025.0159

**Published:** 2026-01-27

**Authors:** Georgia Ramsay, Rachel Brooks, Christina Wade, Jamie Jie Mei Liew, Pavithran Alphonse, Jennifer McIntosh, Laura E Forrest, Jon Emery, Sibel Saya

**Affiliations:** 1 Department of General Practice and Primary Care, University of Melbourne, Melbourne, Australia; 2 Collaborative Centre for Genomic Cancer Medicine, University of Melbourne, Melbourne, Australia; 3 Melbourne School of Population and Global Health, University of Melbourne, Melbourne, Australia; 4 Peter MacCallum Cancer Centre, Melbourne, Australia

**Keywords:** cancer risk, GP perspectives, polygenic risk scores, cancer screening, primary care

## Abstract

**Background:**

A polygenic risk score (PRS) enables personalisation of cancer risk and supporting risk stratification for melanoma, colorectal, breast, and prostate cancers. Including a PRS in a cancer risk assessment can facilitate risk-appropriate cancer screening by incorporating an individual’s age, sex, family history, and genomic test results. GPs are the likely healthcare professionals to order PRS tests and deliver results to patients within existing preventative health models.

**Aim:**

To elucidate GPs’ perspectives on the use of PRSs to tailor cancer screening in the Australian primary care context.

**Design & setting:**

A qualitative study undertaken in Victoria, Australia with GPs involved in a series of studies and clinical trials evaluating PRS.

**Method:**

Thirty GPs were interviewed; they were either PRS naive or had experience of using PRSs in a research context. Participants had a broad spectrum of clinical experience and knowledge of genomics, reflecting the spectrum of experience and knowledge of GPs in Victoria, Australia. Inductive and deductive thematic analysis was conducted and aligned to the Consolidated Framework for Implementation Research.

**Results:**

Common themes identified were: general practice is the appropriate setting for PRS-based approaches, personalised approaches to cancer risk can prompt discussions about positive lifestyle changes, and tailored risk reports are useful tools for the communication of complex health information. Barriers identified by GPs included: time constraints on the delivery of preventative health care, education requirements to upskill GPs in genomics, possible psychosocial harms to patients identified as being at increased risk, life-insurance implications, and added pressure on an already struggling health system.

**Conclusion:**

These findings provide insight into the requirements for the implementation of PRSs in primary care, from the perspective of GPs.

## How this fits in

In research settings, polygenic risk scores (PRSs) are being explored as a strategy for personalised risk assessment and screening recommendations for commonly diagnosed cancers, including melanoma, colorectal, breast, and prostate. Participating GPs, both PRS naive and involved in PRS trials, shared their views on PRS. They saw primary care as the appropriate setting for PRS, fitting within existing preventative health care provided in this setting. Reported barriers included education requirements for GPs and increasing system capacity to care for those identified as being at increased risk. Considering these perspectives is essential for understanding how PRS-based testing could be delivered in primary care.

## Introduction

A polygenic risk score (PRS) can predict an individual’s risk of developing a range of chronic conditions, including some cancers. A PRS applies multiple common single nucleotide polymorphisms (SNPs) that have been identified through genome-wide association studies; each SNP confers a small incremental contribution to an individual’s overall risk of developing a specific disease. PRSs have been developed for a range of diseases including colorectal,^1^ breast,^2^ and prostate cancers,^3^ as well as melanoma,^4^ and can be combined with age, sex, and other risk factors to estimate individual risk in absolute terms and relative to the general population. This approach can risk stratify the population to provide risk-based recommendations for screening activities. For those at greater-than-average risk, increased cancer screening activities (for example: more-frequent screening; commencing screening from an earlier age; or utilising more sensitive, but potentially invasive, screening tests) may be recommended. Conversely, for those with below-average risk, the starting age for screening might be delayed or screening might take place less frequently. Such risk-stratified approaches may be more cost effective than current approaches.^5,6^ The possibility of recommending less screening remains contentious, with clinician^7^ and public^5,8–11^ sentiment on its acceptability and feasibility being mixed.

The potential clinical utility of PRS approaches to risk stratification has been demonstrated in breast,^12^ colorectal,^13^ and prostate cancers.^14^ Ongoing large, randomised trials will provide evidence of clinical and cost effectiveness.^15,16^ A recent systematic review of stakeholder perspectives on PRS-based approaches to risk assessment^17^ highlighted the growing evidence base and showed that much of the published research originates from Australia and the US. Survey-based studies from Canada^18^ and the UK^19^ revealed that primary care physicans (PCPs) and GPs had limited knowledge of PRS-based approaches to breast cancer risk stratification. Participants were concerned about recommending reduced cancer screening activities for those with below-average risk, whereas increasing screening for those at increased risk was seen as a positive application of PRSs.^18,19^ Barriers to implementing PRS use identified by US-based PCPs included cost of testing, the absence of clinical guidelines, and concern about insurance discrimination.^20^ Australian-based research outputs for PRS-based cancer risk assessment have included a number of studies exploring the perspectives of general practice patients^21,22^ or the Australian population;^23^ however, there is limited evidence on the views of GPs, who have been exposed to PRS-based personalised risk information and risk reports for their patients.^7^

Australian-based research is facilitated by support from the Australian Government for evidence-based updates to cancer-screening programmes, and the development of a policy framework for incorporating genomics into cancer control by 2028 is outlined in the *Australian Cancer Plan*.^24^ General practice has been suggested by clinicians as the most appropriate setting for population PRS testing,^25^ with >80% of Australians seeing a GP each year;^26^ indeed, it has been shown that Australians strongly prefer to receive their cancer-risk information in general practice settings.^23^

Education for GPs on these new tests could build on the existing knowledge about genomics from GP-administered prenatal genomic screening and monogenic variant testing for breast and bowel cancer.^27^ In this setting, PRSs could be utilised to extend preventative health through risk stratification, facilitating risk-appropriate screening, and encouraging risk-mitigating behaviour. In this qualitative study, the perspectives of GPs, as the proposed providers of PRS-based risk information and follow-up care, were explored.

## Method

### GP recruitment and consent

GPs were recruited purposively in association with four distinct, sequential research projects, which are outlined in Table 1. The first study explored GPs’ views on PRS use in primary care in the context of preventative health, including cardiovascular disease and cancer; the remaining three projects were sub-studies of trials providing PRS results to patients of interviewed GPs, with individualised cancer risks and associated screening recommendations.^21,22,28,29^ Patient participants in the three trials were encouraged to discuss their report with their GP. The trials utilised a colorectal cancer or a multicancer PRS test (for colorectal, melanoma, prostate, or breast cancers) and provided PRS reports developed to communicate cancer risk and promote risk-appropriate screening. The reports contained the same core information and format, including risk-appropriate screening recommendations along with information about the risks and benefits of the recommended screening test(s).

**Table 1.** Studies in which GPs participated

Study name or acronym	Study type and completion year	GPs, *n*
Implementation of PRS for common disease in Australian general practice	Qualitative study, 2021	Interviews with 10 PRS-naive GPs
CRISP DNA^21,22^	Pilot study of acceptability of a PRS test for colorectal cancer, 2022	Interviews with 12 GPs exposed to a PRS intervention
SCRIPT Trial^28^	Trial of PRS to increase risk-appropriate screening for colorectal cancer risk assessment and screening recommendations, 2023	Interviews with six GPs exposed to a PRS intervention
MAGPIE Study^29^	Trial of cancer PRS including colorectal, melanoma, prostate, and breast cancers, 2024	Interviews with two GPs exposed to a PRS intervention

CRISP = Colorectal cancer RISk Predictor. MAGPIE = Multi-cAncer Genomic risk assessment to target screening in general PractIcE. PRS = polygenic risk score. SCRIPT = SNP Cancer Risk Prediction Trial.

Each study obtained ethical approval from the University of Melbourne Human Research Ethics Committee, and all participants provided written informed consent. With the exception of the first study, the potential pool of GPs was limited to those who had participated in a PRS-based trial.

### Semi-structured interviews

Semi-structured interviews were conducted between March 2019 and November 2023 in person, via videocall, or via telephone call. Interviews were recorded and transcribed, and had a duration of ~30 minutes. Participants who used PRSs in the clinical trials were asked to share their experience of using the report with their patients, including their opinions on report content and presentation. Interview schedules (see Supplementary Information S1) addressed domains of the Consolidated Framework for Implementation Research (CFIR) to support exploration of the multidimensional nature of implementation barriers and facilitators.^30^

### Qualitative analysis, including reflexivity

Reflexive thematic analysis, with a mixed inductive and deductive approach, was used to analyse the interview data.^31^ Interviews were conducted by five authors in their capacity as a research assistant, Master’s student, or PhD student. All interviewers had regular supervision to discuss and reflect on the content raised, the context of the interview (within an overarching trial or with GPs who were PRS naive), and the approach of the interviewer themselves.

One or two of the five authors mentioned took the lead for the inductive coding and initial grouping into broad categories for each of the four interview groups. Initial coding of the interviews was conducted inductively by systematically reading and rereading the transcripts and coding each concept that interviewees discussed. Coding within each sub-study occurred while data collection was taking place to facilitate the iterative additions to, and refining of, the interview schedules. Following completion of the initial coding for each sub-study, similar codes within each sub-study’s interviews were grouped into themes. These themes were then categorised deductively into the following CFIR domains:

- intervention characteristics;
- individual characteristics;
- inner setting;
- outer setting; and
- implementation process.^30^

To combine codes and themes across the four sub-studies, one researcher identified commonalities and differences between the sub-studies, and refined the themes with a focus on relevance to the research question and the CFIR domains. A subset of interviews (at least 10%) in each of the four groups underwent co-coding to support consistency, with coding discrepancies discussed and resolved through consensus. The researchers who were involved in the coding also discussed how their varied professional backgrounds and experience impacted their coding choices, ensuring reflexivity was addressed in the analysis process. Some themes spanned two domains; in those cases, a consensus approach was used to decide whether a single domain or two domains was most appropriate. Two authors were involved in all projects, ensuring consistency and knowledge of the context in which the interviews took place. NVivo (version 14) software was used to organise the codes and themes.

## Results

Thirty GPs based in Victoria, Australia, participated in an interview; their characteristics are given in Table 2. In total, 10 GPs participated in a study exploring the broad use of genomics in primary care (described as ‘PRS-naive GPs’), 12 in the pilot clinical trial using a PRS test for colorectal cancer screening, six in a randomised controlled trial using a PRS test for colorectal cancer screening, and two in a pilot trial for a multicancer (melanoma, colorectal, breast, and prostate cancer) PRS test. Of the 72 GPs who consented in these trials, 20 completed a post-trial interview. Barriers to recruitment included the impact of COVID-19 on GP capacity and GPs having left the clinic where the trial took place.

**Table 2.** Participants’ characteristics, *n* = 30

Characteristic	*n* (%)
**Gender**	
Woman or female	16 (53.3)
Man or male	14 (46.7)
**Years of experience in general practice**	
1–9	9 (30.0)
10–19	6 (20.0)
20–29	5 (16.7)
30–39	3 (10.0)
≥40	5 (16.7)
Missing	2 (6.7)
**Clinic location**	
Metropolitan	28 (93.3)
Rural	2 (6.7)

Themes categorised by the CFIR domains are summarised in Table 3.

**Table 3.** Results summary: themes of GP perspectives on the use of PRS for cancer in general practice, categorised by Consolidated Framework for Implementation Research domains

Consolidated Framework for Implementation Research domain				
**Intervention characteristics**	**Individual characteristics**	**Inner setting**	**Outer setting**	**Implementation process**
GPs want evidence-based guidelines for using PRS GPs exposed to PRS in a trial context view content and format as useful for communicating risk to their patients Risk of PRS results causing distress PRS may encourage health-promoting behaviour change	Varied understanding and experiences with genetic or genomic testing Focus on preventative health GP perception of patients’ willingness to make healthy lifestyle changes GPs need education about PRS before implementation can occur	General practice as the appropriate setting for PRS use Relative priority of preventative health activities when patients usually present when unwell Limitation of appointment duration on providing thorough explanation of PRS	Risk of referral bottlenecks if many patients are classified as being at increased risk Pressure on GPs to fit increasingly more into ‘standard consultations’ Insurance implications Risk of exacerbating health inequities	General practice as the appropriate setting for PRS Evidence-based, endorsed guidelines (with clear referral pathways for those at increased risk) are essential Equitable access to PRS with minimal or no out-of-pocket expense for patients

PRS = polygenic risk score;

### Appropriateness of the primary care setting (inner setting)

Most GPs thought that general practice settings were the appropriate location for using PRSs. As one participant stated:

*‘If there is genomic testing that is shown to be cost effective that is supported by a wraparound health service, then a GP is actually best placed to coordinate that.’* (GP01, PRS naive)

The incorporation of PRSs into cancer prevention and screening was reported to align with the preventative health care already being provided by GPs:

*‘We’re seeing people for routine check-ups and you’re thinking all the preventative health stuff, I have to check their cholesterol, have we done this, have we done that, that might be the time to raise something like this.’* (GP11, PRS naive)

Many GPs thought that genomic testing, including PRSs, was something that will inevitably be integrated into routine care:

‘*I could see it become more part of regular screening for other things* [beyond colorectal cancer] *as well.’* (GP27, in PRS trial)

*‘I think that’s probably the way, the future of medicine to be honest.’* (GP28, in PRS trial)

Some GPs noted that patients aged 45–49 years were eligible for a health check, covered by a specific government rebate, and considered this a possible opportunity to integrate PRS tests into existing preventative health structures:

*‘… they say 45 is the time most problems start so they come in for general check-ups … ’* (GP17, in PRS trial)

### Health-promoting behaviour change (intervention characteristics)

Some GPs thought PRS information could be useful in encouraging patients to engage with health-promoting lifestyle modifications:

*‘… well you say, “Okay, you're okay for prostate cancer and bowel cancer, but you still have that smoking situation”.’* (GP30, in PRS trial)

*‘… you can say, “Well, actually, you come out high on the genetic score, so I would suggest you change your diet and I would suggest you stop smoking and we’re going to do a colonoscopy on you”.’* (GP15, in PRS trial)

However, a few GPs were less optimistic about the role of PRS in facilitating positive lifestyle changes to reduce cancer risk:

*‘You would have to frame the risk* [to show] *that it was significant …* [O]*besity was associated with an increased incidence but we know that. Everybody’s been publicising, there are about 10 cancers that obesity is a known risk factor* [for]*, but nothing’s changed.’* (GP16, in PRS trial)

### Consultation limitations (inner setting and outer setting)

The short duration of standard consultations and the rarity of patients presenting purely for preventative health was commonly reported as a barrier to PRS implementation:

*‘I think as a GP you have to be very motivated about* [preventative health] *in order for the patients to get really excited and motivated as well to actually do it.’* (GP24, in PRS trial)

Many GPs also reported providing cancer screening advice or updating family history only after they had addressed the primary reason for the patient’s presentation.

The prospect of needing to provide counselling and/or arrange investigations or a referral to secondary care within standard duration appointments for patients who were undergoing genomic testing was concerning for some GPs:

*‘…* [this] *often takes more … than the allocated 15 minutes, just to see where they're coming at with it, what experiences they’ve had with a relative or a friend. Things where I'm not necessarily referring them onto the service but trying to work out if there are risk factors to warrant sending them on.’* (GP29, in PRS trial)

### GPs’ educational and resource needs (intervention characteristics and implementation process)

Although most interviewees viewed genomic tests as becoming a component of personalised medicine in the future, they reported requiring additional education to further their understanding of genomics and how PRS-based approaches could be integrated into routine practice:

*“*[A] *lot of education and training will be required because you have a huge cohort of GPs who are at different stages of their careers … I work with colleagues here coming towards the tail end of their career and so this didn't even exist in terms of medicine whenever they were coming through.’* (GP28, in PRS trial)

Many responders highlighted the need for guidelines endorsed by a governing body before any significant practice change could be made, which would reassure GPs about the value of using PRS-based approaches:

*‘I would want to see national guidelines … some synthesised sort of evidence, what the state of play was, how it might be useful in general practice, and what resources would be available.’* (GP07, PRS naive)

*‘I'd want to know what the guidelines are about who should have the testing, when and how, etc …* [a]*nd then I would use it according to guidelines.’* (GP23, in PRS trial)

### Referral pathways (implementation process)

Several responders voiced concern about how increased cancer screening activities for those identified as being at increased risk for a type of cancer — for example, screening via colonoscopy every 5 years rather than having a biennial immunochemical faecal occult blood test — could, potenitally, burden the health system:

*‘… it would actually bring more burden into healthcare or it would help? I’m not sure.’* (GP21, in PRS trial)

*‘So I'd be very wary that it could potentially squeeze things in maybe the tertiary centres, perhaps, but then what happens at the bottleneck?’* (GP29, in PRS trial)

### Equitable access to preventative health (implementation process)

Although some GPs thought patients might be willing to pay an out-of-pocket fee to receive an individualised PRS, many expressed concern about the cost of testing and the risk of those of lower socioeconomic status missing out, thereby amplifying health disparities:

*‘… on a population level, it’s tricky … the people who want to do preventative health activities will do them, and those who don't want to do them, or because of other socioeconomic barriers, will still proceed to miss out as a result of it.’* (GP28, in PRS trial)

Many interviewees highlighted the need for evidence to support the use of PRSs in the Australian setting through cost–benefit analyses of PRS-informed risk-stratified screening:

*‘… you’ve got to hang in there for the next 10 years and see how many of the people get cancer, you know, before you can actually say “yes it did, in fact, save lives”. And that’s what governments want to know. And, what’s more, governments actually want to know if it saves them money.’* (GP15, in PRS trial)

They stated that the government should fund such a test only if there was sufficient evidence to justify the use of PRSs to inform screening, removing the need for patients to pay out of their own pockets:

*‘… it would definitely have a role if it was cost effective as well, and was covered by the government … I think cost is a huge influence in whether a patient decides to opt for that investigation. And the doctors as well, as a result.’* (GP21, in PRS trial)

### Distress caused by PRS results (intervention characteristics)

Some GPs expressed concern about PRS results being a source of anxiety for some patients, especially those stratified into higher risk categories:

*‘… the downside would be that it does cause more patients anxiety when people think they're at higher risk, and people’s understanding of risk varies.’* (GP23, in PRS trial)

Although some GPs saw pre- and post-test counselling as falling within their scope of practice, others thought that access to genetic counselling services would be an essential part of the implementation process:

*’I'm sure there’s not all that many genetic counsellors on the ground. So that’s going to be the problem is what to do with the results when they come back.’* (GP30, in PRS trial)

However, some GPs reported that their experience of patients who had an increased risk identified from a PRS did not display significant anxiety, partly due to there being clear recommendations for risk-appropriate screening activities in order to manage the risk:

*‘I think that* [distress] *can be contained because, if they do have increased risk, they will probably be advised to have the colonoscopy … so I think those concerns were probably less than they were, having seen* [the report]*.’ (*GP14, in PRS trial)

Importantly, this sentiment endured for GPs who were exposed to the multicancer PRS trial reports, where patient-participants were informed that they were at above-average risk for up to three cancers (melanoma, colorectal, and breast or prostate cancer):

*‘I think I was impressed with the lack of anxiety that the slightly increased risk group displayed when they came* [to see me] *… Like whether the counselling had been quite good, or you know, they were kind of not, they weren’t, no one came back paranoid … Which was a bit of a worry with the test, I was worried that would happen. I was worried that I would get some people who it could potentially tip their health anxiety over the edge.’* (GP29, in PRS trial)

### Insurance implications (outer setting)

Concerns about insurance implications — especially for people who return greater-than-average risk results — was common:

*‘I think it’s knowing if that person has an increased risk, what an insurance company might do with that in terms of premiums and whether … they should disclose that or not.’* (GP14, in PRS trial)

*‘If I was a 20-year-old professional, I would be not doing the test ‘til I've got income protection and so forth.’* (GP29, in PRS trial)

However, no participants reported knowledge of the Australian Memorandum on Genetic Tests in Life Insurance.^32^

### Communication tools (intervention characteristics)

**Figure 1.**
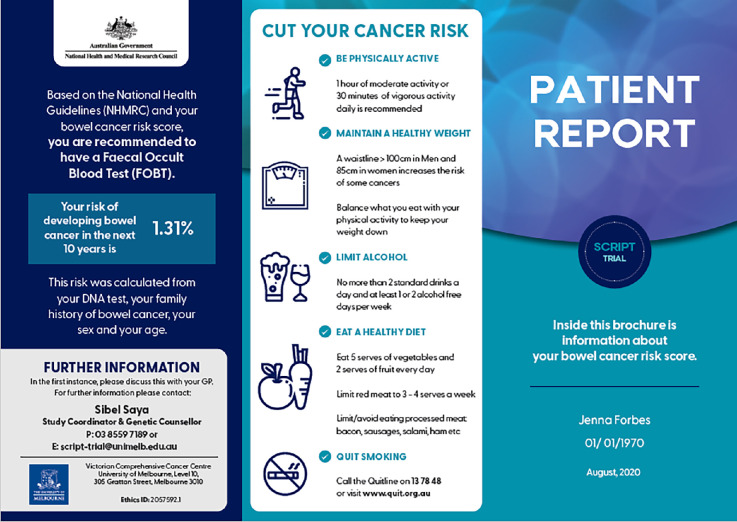
Example of a polygenic risk score report for colorectal cancer risk used in the SCRIPT Trial.^28^

**Figure 2.**
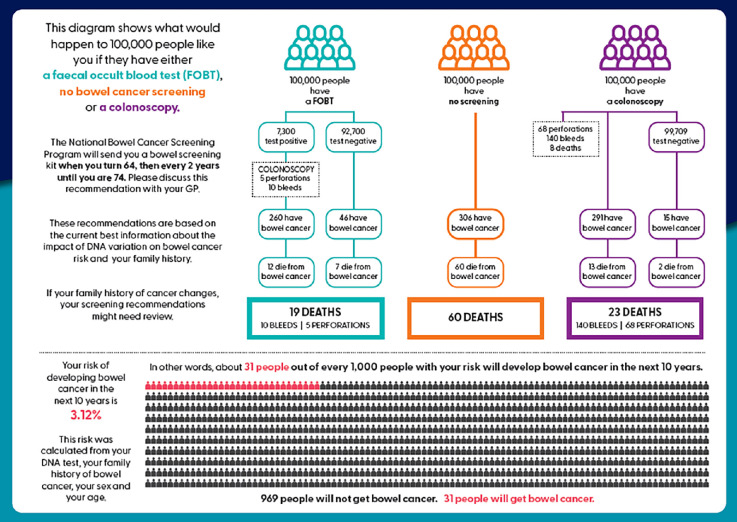
Example of polygenic risk score report risk communication for personalised colorectal cancer risk.^28^

Of the 30 participants, 18 were exposed to a PRS report for their patients in the context of a research study. Figures 1 and 2 show an example of the personalised risk report provided to participants (both patients and GPs).

Some of those GPs indicated how useful the PRS reports could be:

*‘*[B]*ased on my normal conversation with people* [about colorectal cancer screening] *… if I don’t have this kind of visual representation and just based on my, I suppose, verbal education to them, or whatever, maybe there’s a less of an uptake. So I think certainly, this is a useful tool to guide their decision making.’* (GP19, in PRS trial)

Additionally, the design of the report, with reinforcement of screening recommendations by their GP, resulted in patients clearly understanding next steps:

*‘… the report, I think, must have been very good and readable, because I think that’s why the patients weren’t particularly anxious for themselves, and as long as I was endorsing that* [same screening recommendation]*, that’s why they were probably quite relaxed.’* (GP29, in PRS trial)

The report was a conversation starter and, in the context of colorectal cancer screening, reduced discomfort around subjects such as bowel function and faecal sample collection:

*‘I think it probably made them more comfortable to talk about it … I think that was definitely an easier approach.’* (GP27, in PRS trial)

## Discussion

### Summary

GPs in this study foresaw genomics being integrated into their future practice and considered general practice to be the most appropriate setting for PRS-based approaches. Some also acknowledged the potential for returning personalised risk information to motivate patients to positively modify their behaviour. GPs also identified several barriers to implementing PRSs, including the need for education and training, evidence-based guidelines to inform appropriate PRS use, and timely access to secondary care for those identified at increased risk of cancer. Availability of psychosocial support for individuals at increased risk and the limited opportunities for preventative health within standard duration appointments were important implementation considerations.

### Strengths and limitations

Self-selection of interview participants and GP clinics for participation in the overarching trials may have introduced a bias towards GPs with an interest in genomics, cancer screening, or preventative medicine. In addition, GPs’ knowledge of PRSs is likely to have evolved over the 5 years of data collection; although this study was not designed to explicitly explore this, evaluating GP perspectives over a period of 5 years in serial studies and demonstrating enduring themes provides useful information for policymakers and those involved in the implementation of risk-stratified cancer screening programmes.

It is important to note the small sample size of 30 GPs. They were primarily delivering care in Victorian metropolitan settings meaning the perspectives of GPs practising in remote or regional Australia are under-represented. Generalisibility of findings must therefore be made with caution. Another limitation is that most participating GPs had experience with PRS reports by receiving individualised reports for their patients involved in research, which does not represent the experience of most GPs. However, this allowed for an exploration of how GPs’ exposure to PRSs affected their perspectives. The commonality in themes expressed by both groups — that is, those who were PRS naive and those who were not — and the diversity in knowledge and experience of genomics among Australian GPs suggests that barriers identified in this study require consideration before the introduction of routine PRS use in general practice.

Although this study includes the perspectives of Australian-based GPs, findings are useful in understanding factors that support and impede the implementation of PRS-based approaches in other countries, particularly those with comparable health systems.

### Comparison with existing literature

The findings presented here on the use of PRSs in colorectal cancer and multicancer tests are aligned with related work exploring Australian GPs’ perspectives on PRS use for melanoma, in which GPs conveyed the need for practice guidelines to underpin any change to clinical practice and the importance of preventing psychological harms.^33^ Although concerns were raised regarding possible referral bottlenecks, GPs should engage in patient-centred care and use clinical judgement that includes balancing preventative health activities with other patient priorities. A US study demonstrated that primary care providers consider a patient’s individual circumstances before deciding to change their clinical management based on genomic results.^34^ Although numerous participants expressed concern about possible psychological harm arising from genomic testing, several reflected that this did not occur for their patients who were identified as being at increased cancer risk in the PRS trial in which they had participated. The level of concern expressed by GPs was inversely related to level of previous exposure to PRS, particularly when their patients were stratified into above-average-risk categories. This is concordant with findings from a systematic review of studies providing PRS results to individuals, which demonstrated limited enduring psychosocial impact of receiving PRS information.^35^

Insurance implications of genomic testing for risk stratification were also a concern reported by GPs and reflects a broader discourse in Australia about how genomic test results should be used by insurers to limit the value or type of coverage people can obtain.^32^

### Implications for research and practice

Educational strategies and clinical guidelines may improve GP capacity to provide PRS-guided care and inform how to better manage those with high-risk PRS results, including referral to specialty genetics services as required. The findings presented here showed that GPs identified that appropriate referral pathways and health-system capacity should be considered with the implementation of PRS use in general practice. Genomic information could be used by GPs as an additional tool in the decision-making process, rather than something to replace clinical decision making. For multicancer PRS approaches, it will be important both to obtain estimates of the number of individuals that will be categorised into higher-risk groups across multiple cancer types and to develop appropriate referral pathways for those with more-complex risk profiles.

PRS-based approaches to cancer risk differ from monogenic approaches that have been the major focus of genetics services, suggesting that preparedness of clinical geneticists, genetic counsellors, and others involved in care may be lacking. Evaluation of Australian clinicians’ preparedness to expand their practice to include PRSs indicated that even those with a comparatively high level of relevant genomics knowledge felt only ‘somewhat prepared’ (45.7%) or ‘not at all prepared’ (43.8%).^36^ This highlights the importance of whole-system strategies to prepare for the use of PRS-based cancer risk assessments and associated risk-informed cancer screening encompassing education for GPs, clinical geneticists, genetic counsellors, and others across the health sector.^37,38^

Although evidence suggests most individuals who receive genomic-informed risk results will not experience psychological harm,^35^ pathways must be developed to ensure that those who do receive appropriate and timely support. The option for a telephone call with a genetic counsellor has been tested in the context of melanoma PRSs in Australia, with participants reporting a high level of satisfaction.^39^ This may provide a suitable option for individuals requiring support beyond what can be delivered by their GP. Concerns around psychological safety also highlight the importance of how genomic risk is communicated to patients. Providing risk information in multiple formats (for example, verbally or via graphs and icon arrays) facilitates effective content communication for people with varied health literacy;^40^ this approach is being used in colorectal^28^ and multicancer contexts.^29^

There is the potential for PRS-based approaches to provide opportunities to encourage appropriate screening activities, including for those people at average risk of developing cancer, and protective lifestyle modifications. Internationally, there is mixed evidence on the behavioural impact (increasing risk-appropriate cancer screening) of personalised risk estimates alone,^35,41,42^ but compelling arguments have been made for trialling risk assessments that have been integrated with proven and behavioural-theory-based interventions.^35,43^ The value of GP endorsement of cancer screening has been shown, internationally, to increase uptake of breast^44^ and colorectal cancer screening.^45^ Although individuals may not act on this advice, primary care clinicians suggest that recommendations regarding cancer prevention may be more impactful than prevention for other chronic conditions.^25^ This may present an opportunity to increase participation in Australian population screening programmes, which currently report participation rates as being 52% for breast cancer^46^ and 41% for colorectal cancer.^47^ Additionally, PRS approaches that include age, sex, and family history, require up-to-date family history details that can lead to the identification of people who should be offered increased screening options, referral to familial cancer services, and testing for high-risk monogenic variants, regardless of their PRS results.^48^

Currently, a moratorium preventing insurance companies from using genomic test details for risk-rating life insurance policies to monetary thresholds exists in Australia.^49^ Further announcements regarding a permanent, forthcoming ban on insurers using genetic test results in this manner are expected;^50^ this necessitates further work to reassure patients and their treating clinicians that genomic testing will not result in insurance issues and safeguards to ensure insurers comply with their obligations.^51^

There is consistent GP support for their involvement in using PRSs to support risk-stratified cancer screening. This spans from GPs with little prior exposure to genomic testing to those who have experience through PRS-based cancer risk reports in a clinical research context. Future research is needed to test scalable models of the implementation of PRS in general practice. This needs to occur while policy frameworks for risk-stratified cancer screening are developed.^24,52^ Consideration of how to refine existing screening programmes to include evidence-based, cost-effective changes in an already stretched health system is essential.

This work is directly informing current clinical trials of multicancer PRS use in general practice in terms of resources and support provided for participating GPs, and strategies for communicating risk to both GPs and patients. As highlighted by Skivington *et al*,^53^ acting on this feedback and adapting the multicancer PRS intervention to meet the needs of end users in general practice is an essential step in ensuring its suitability within this environment. A randomised controlled trial^16^ aiming to provide further evidence for the clinical utility and cost-effectiveness of using PRSs to tailor cancer screening in Australian general practice is currently under way.

## Data

The data are not publicly available as they contain information that could compromise the privacy of research participants.
